# Type 2 diabetes is more predictable in women than men by multiple anthropometric and biochemical measures

**DOI:** 10.1038/s41598-021-85581-z

**Published:** 2021-03-15

**Authors:** Tangying Li, Huibiao Quan, Huachuan Zhang, Leweihua Lin, Lu Lin, Qianying Ou, Kaining Chen

**Affiliations:** 1 Department of Health Care Centre, Hainan General Hospital, Hainan Affiliated Hospital of Hainan Medical University, Haikou, 570311 Hainan China; 2 Department of Endocrinology, Hainan General Hospital, Hainan Affiliated Hospital of Hainan Medical University, No.19 Xiuhua Road, Haikou, 570311 Hainan China; 3 Department of Endocrinology Laboratory, Hainan General Hospital, Hainan Affiliated Hospital of Hainan Medical University, Haikou, 570311 Hainan China

**Keywords:** Type 2 diabetes, Predictive markers

## Abstract

Men and women are sexually dimorphic but whether common anthropometric and biochemical parameters predict type 2 diabetes (T2D) in different ways has not been well studied. Here we recruit 1579 participants in Hainan Province, China, and group them by sex. We compared the prediction power of common parameters of T2D in two sexes by association, regression, and Receiver Operating Characteristic (ROC) analysis. HbA1c is associated with FPG stronger in women than in men and the regression coefficient is higher, consistent with higher prediction power for T2D. Age, waist circumference, BMI, systolic and diastolic blood pressure, triglyceride levels, total cholesterol, LDL, HDL, fasting insulin, and proinsulin levels all predict T2D better in women. Except for diastolic blood pressure, all parameters associate or tend to associate with FPG stronger in women than in men. Except for diastolic blood pressure and fasting proinsulin, all parameters associate or tend to associate with HbA1c stronger in women than in men. Except for fasting proinsulin and HDL, the regression coefficients of all parameters with FPG and HbA1c were higher in women than in men. Together, by the above anthropometric and biochemical measures, T2D is more readily predicted in women than men, suggesting the importance of sex-based subgroup analysis in T2D research.

## Introduction

High plasma glucose levels can cause diabetes mellitus, which can lead to clinical complications including cardiovascular morbidities, renal impairment, retinopathy. Type 2 diabetes (T2D) can result from the dysfunction of insulin-secreting β-cells, the resistance of insulin by peripheral tissues such as muscle, or both^1,2^. T2D is currently diagnosed by fasting plasma glucose (FPG), hemoglobin A1c and the oral glucose tolerance test (OGTT)^3^. But these factors remain insufficient in predicting the development of diabetic complications^4,5^. Therefore, efforts have been focused on identifying better predictors for T2D. Many anthropometric and biochemical markers have been investigated, including BMI, body adiposity index (BAI), waist circumference and waist-to-hip ratio, waist-to-height ratio, high blood pressure, triglyceride levels, insulin and proinsulin levels, and proinsulin-to-insulin ratio (P/I ratio). However, some studies remain inconsistent and conclusive results have not been reached.

Men and women are different in many ways; however, other than a few diseases related to the reproduction system such as breast cancer and testicle cancers, the sex difference in many prevalent diseases has not been carefully evaluated^6^. T2D is one of the most prevalent diseases that also show sexual dimorphism in many aspects, including diagnosis, prevention, and treatment^7,8^. For example, men have a higher risk of T2D and are diagnosed at a younger age^9–12^. However, women with T2D are prone to develop more severe complications than men^8,13^. Women with T2D have a higher risk for coronary heart disease than men with T2D^14–16^. The sex difference in T2D has been attributed to both biological and psychosocial factors^7^.

Several anthropometric and biochemical parameters are associated with T2D differently between men and women. For example, in a study on middle-aged Caucasians, low vitamin D3 was independently associated with T2DM in women but not in men^17^. In a pre-diabetes cohort of East Asians, age, FPG, triglyceride levels, and smoking status were found to be associated with T2D development stronger in men while waist circumference was found to be associated with T2D development only in women^18^. In multiple studies, men were diagnosed with T2D at a lower BMI^19–21^. HbA1c predicted T2D better in women in a French cohort^22^. Despite these interesting studies, a systematic comparison between the two sexes has not been reported.

In this study, we analyzed a cohort of 1579 East and Southeast Asians from Hainan Province, China, comparing between women and men the association and prediction power of multiple anthropometric and biochemical parameters. Our study provides novel knowledge regarding the sex-difference in T2D prediction and prevention.

## Results

### HbA1c predicts T2D better in females than in males

The 1579 participants were grouped into 567 men and 1012 women. Men had slightly higher T2D cases (N = 93) compared to women (N = 128). Complications related to cardiovascular diseases and diabetic complications were rare (Table S1). The cases of diabetic medicine use were 23 in men and 24 in women, making no significant difference between both groups by Chi-square test. However, the group of men had a significantly higher representation of overweight (BMI > 25 kg/m^2^), smoking, hyperlipidemia, and hyperuricemia (Table S1), which were used to adjust the association and regression studies.

We first compared the difference of multiple physical and chemical characteristics in males and females. The mean age of males was slightly older than females, with a mean ± standard deviation of 49.16 ± 13.03 vs 47.43 ± 13.5, respectively. Fasting plasma glucose was not significantly different between males and females (5.56 ± 1.70 in males vs 5.43 ± 1.28 in females). The percentage of HbA1c was slightly higher in males (5.78 ± 1.03 in males vs 5.66 ± 0.92 in females, P = 0.02). Fasting and glucose-stimulated insulin levels did not differ significantly between males and females (66.82 ± 47.17 pmol/L in males vs 66.13 ± 51.57 pmol/L in females and 502.53 ± 458.33 pmol/L in males vs 496.80 ± 443.29 pmol/L in females, respectively). Fasting and glucose-stimulated proinsulin were significantly higher in males (17.13 ± 17.18 vs 12.17 ± 9.82 pmol/L and 73.50 ± 64.31 vs 56.50 ± 44.91 pmol/L, respectively). All other physiological and biochemical measurements showed a significant difference between the two sexes (Table S1).

**Figure 1:**
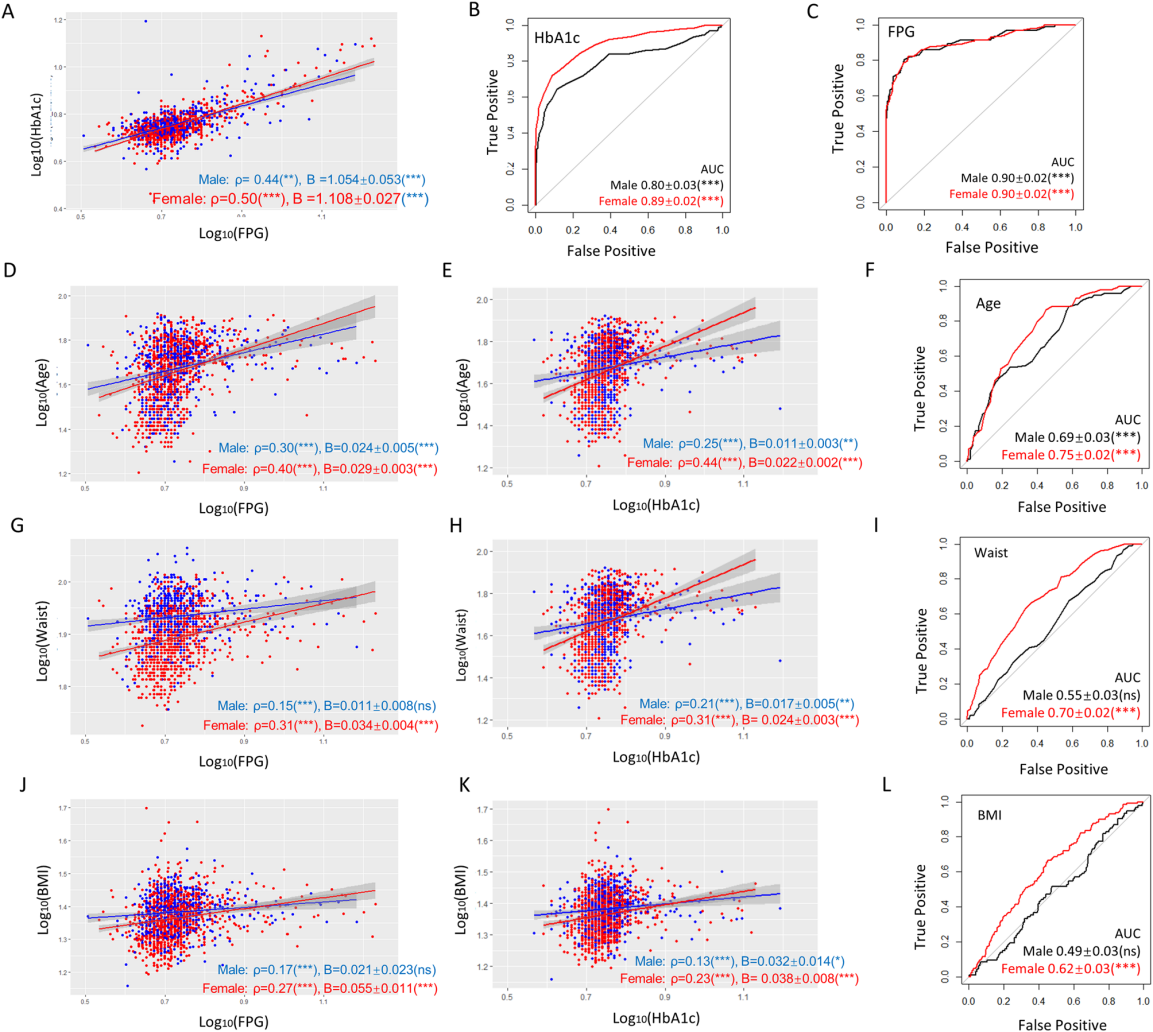
HbA1c, Age, waist circumference and BMI predict T2D better in women than in men. (**A**) Comparisons of association and linear regression of HbAc1 to FPG in women and men. Spearman’s association coefficient (ρ) indicates a tighter association in women. Linear regression coefficient (**B**) indicates a stronger influence of HbA1c on FPG levels in women. P values, regression constant can be found in Tables S2 and S3. (**B**) HbA1c is a better T2D predictor in women. Receiver operating characteristic (ROC) was plotted for HbA1c and T2D and area under the curve (AUC) is indicated. (**C**) ROC-AUC analysis shows no sex-difference of FPG in predicting T2D. (**D**,**E**) Age has a tighter association (Spearman’s ρ) with and stronger influence (steeper slope) on FPG and HbA1c in women. (**F**) Age is a stronger T2D predictor for women (higher ROC-AUC values). (**G**,**H**) Waist circumference has a tighter association with and stronger influence on FPG and HbA1c in women. (**I**) Waist circumference is a stronger T2D predictor for women as judged by ROC-AUC. (**J**,**K**) BMI has a tighter association with and stronger influence on FPG and HbA1c in women. (**L**) BMI predicts T2D in women but not men as judged by ROC-AUC analysis. *AUC* area under the curve, *ns* not significant, *P < 0.05, **P < 0.01, ***P < 0.001.

Next, we were interested in knowing the sex difference regarding the correlation of HbA1c and FPG. As shown in Fig. 1A, the Spearman correlation coefficient was higher in females than in males (ρ = 0.5 vs 0.44, P < 0.01). We also did linear repression of HbA1c and FPG. The coefficient in females was higher than in males (1.054 ± 0.053 vs 1.108 ± 0.027, P < 0.001). Consistently, binary logistic regression analysis demonstrated that HbA1c showed a much steeper regression slope with FPG in women than in men (3.071 ± 0.262 vs 1.450 ± 0.199, P < 0.001, Table 1). This trend remained true after adjusting for age, smoking, overweight, hyperlipidemia, hyperuricemia, and T2D. To confirm this idea, we analyzed the data with receiver operating characteristic (ROC) and measured area under the curve (AUC) in both men and women. ROC-AUC values demonstrated that HbA1c was a much stronger predictor of T2D in women than in men (0.89 ± 0.02 vs 0.80 ± 0.03, P < 0.001, Fig. 1B). In contrast, FPG has no difference in predicting T2D in men and in women (Fig. 1C). Consistently, binary logistic regression demonstrated that HbA1c but not FPG showed a higher association with T2D in women than in men even after adjustment (Table 1).

**Table 1:** Binary logistic regression analysis of anthropometric and biochemical parameters for T2D.

Parameters	Men				Women			
	Constant	B ± SD	P	Exp(B)	Constant	B ± SD	P	Exp(B)
FPG (mmol/L)	− 16.255	2.532 ± 0.268	0.000	12.578	− 16.272	2.489 ± 0.213	0.000	12.046
HbA1c (%)	**− 10.232**	**1.450 ± 0.199**	**0.000**	**4.262**	**− 20.115**	**3.071 ± 0.262**	**0.000**	**21.560**
HbA1c adjusted^a^	**− 12.80**	**1.271 ± 0.192**	**0.000**	**3.564**	**− 21.108**	**2.831 ± 0.268**	**0.000**	**16.960**
Age (years)	− 4.697	0.059 ± 0.01	0.000	1.060	− 5.607	0.071 ± 0.008	0.000	1.073
Waist circumference (cm)	− 3.345	0.020 ± 0.013	0.134	1.020	− 8.466	0.081 ± 0.011	0.000	1.084
BMI (kg/m^2^)	− 1.786	0.007 ± 0.037	0.857	1.007	− 3.996	0.087 ± 0.023	0.000	1.091
Systolic pressure (mmHg)	− 3.811	0.017 ± 0.006	0.006	1.017	− 5.285	0.027 ± 0.004	0.000	1.027
Diastolic pressure (mmHg)	− 2.200	0.007 ± 0.009	0.433	1.007	− 4.332	0.031 ± 0.008	0.000	1.031
Triglyceride (mmol/L)	**− 1.794**	**0.071 ± 0.042**	**0.089**	**1.074**	**− 2.394**	**0.251 ± 0.068**	**0.000**	**1.285**
Triglyceride adjusted^a^	**− 5.855**	**0.086 ± 0.042**	**0.050**	**1.090**	**− 6.101**	**0.166 ± 0.065**	**0.011**	**1.181**
Total cholesterol (mmol/L)	**− 2.219**	**0.107 ± 0.108**	**0.323**	**1.113**	**− 4.342**	**0.431 ± 0.081**	**0.000**	**1.538**
Total cholesterol adjusted^a^	**− 5.900**	**0.058 ± 0.114**	**0.613**	**1.059**	**− 7.066**	**0.218 ± 0.089**	**0.014**	**1.243**
LDL (mmol/L)	**− 1.621**	**− 0.001 ± 0.132**	**0.994**	**0.999**	**− 3.562**	**0.535 ± 0.111**	**0.000**	**1.708**
LDL adjusted^a^	**− 5.446**	**− 0.044 ± 0.137**	**0.748**	**0.957**	**− 6.690**	**0.261 ± 0.123**	**0.033**	**1.299**
HDL (mmol/L)	**− 1.427**	**− 0.145 ± 0.4**	**0.717**	**0.865**	**− 0.451**	**− 0.968 ± 0.327**	**0.003**	**0.380**
HDL adjusted^a^	**− 4.995**	**− 0.457 ± 0.451**	**0.310**	**0.633**	**− 4.091**	**− 1.281 ± 0.363**	**0.000**	**0.278**
Fasting insulin (pmol/L)	− 2.070	0.006 ± 0.002	0.003	1.006	− 2.640	0.010 ± 0.002	0.000	1.010
Fasting proinsulin (pmol/L)	− 2.359	0.037 ± 0.007	0.000	1.038	− 2.734	0.057 ± 0.009	0.000	1.059

^a^Adjusted for age, smoking, overweight, hyperlipidemia, hyperuricemia and diabetes.

Items in bold indicate significantly different odd ratios Exp(B) between men and women.

### Age, waist circumference and BMI predict T2D better in females than in males

We continued to examine other anthropometric and biochemical parameters for their correlation and regression pattern with FPG and HbA1c, as well as their prediction strength for T2D. As shown in Fig. 1D,E,G,H,J,K, age, waist circumference, and BMI showed a stronger association with FPG and HbA1c in women than in men, as judged by Spearman association coefficient (ρ). In general, the stronger association in women remained true after adjustment for age, smoking, overweight, hyperlipidemia, hyperuricemia and T2D (Table S2). Linear regression between these parameters with FPG and HbA1c showed a steeper slope and higher coefficient (B) in women than in men (Fig. 1 and Table 1). The ROC-AUC values were all higher in women than in men: 0.75 ± 0.02 (P < 0.001) vs 0.69 ± 0.03 (P < 0.001) by age, 0.70 ± 0.02 (P < 0.001) vs 0.55 ± 0.03 (ns, not significant) by waist circumstance, and 0.62 ± 0.03 (P < 0.001) vs 0.49 ± 0.03 (ns) by BMI (Fig. 1F,I,L), indicating that they could better predict T2D progression in women than in men. Consistently, binary regression to T2D showed higher coefficients in women than in men (Table 1).

### Blood pressure predicts T2D better in females than in males

**Figure 2:**
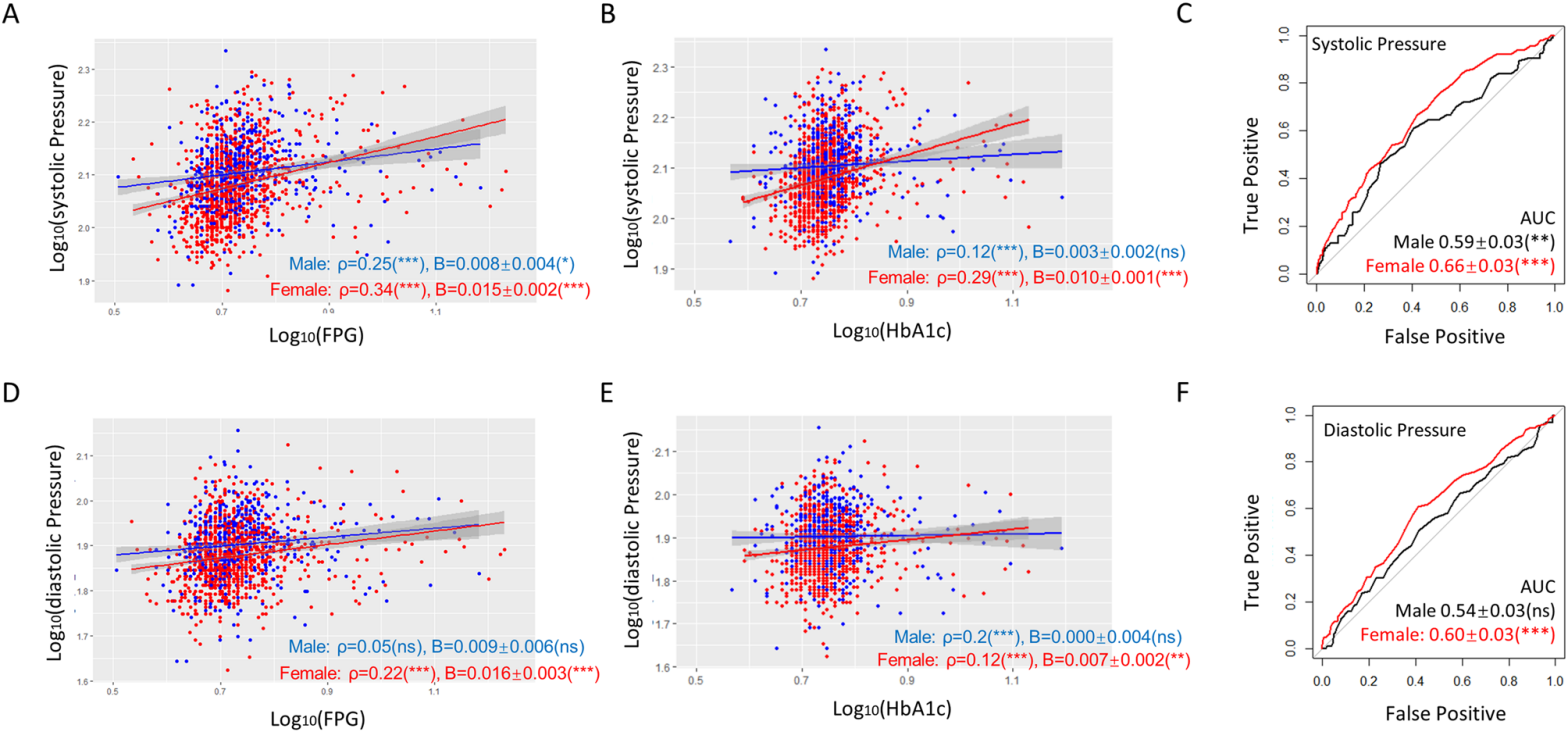
Blood pressure predicts T2D better in women than in men. (**A**,**B**) Systolic blood pressure has a tighter association (Spearman’s ρ) with and stronger influence (steeper slope) on FPG and HbA1c in women. P values, regression constant can be found in Tables S2 and S3. (**C**) Systolic blood pressure is a stronger T2D predictor for women as judged by ROC-AUC analysis. (**D**) Diastolic blood pressure tends to have a tighter association with FPG and a stronger influence on FPG in women than in men. (**E**) Diastolic blood pressure has a tighter association with HbA1c in men but tends to have a stronger influence on women. (**F**) Diastolic blood pressure is a weak T2D predictor for women and has neglectable prediction power for men. *AUC* area under the curve, *ns* not significant, *P < 0.05, **P < 0.01, ***P < 0.001.

As shown in Fig. 2, systolic blood pressure was associated with FPG (ρ = 0.34 vs 0.25) and HbA1c (ρ = 0.29 vs 0.12) stronger in women than in men. Diastolic blood pressure was less associated with HbA1c in women than in men (ρ = 0.12 vs 0.20). However, linear regression demonstrated that both systolic and diastolic blood pressure influence FPG and HbA1c stronger in women than in men, as the slopes were steeper in all cases (Fig. 2A,B,D,E). These trends remained true even after adjustment for confounding factors including age, smoking, overweight, hyperlipidemia, hyperuricemia, and T2D (Table S2).

Despite the generally weak prediction power, both systolic and diastolic blood pressure could predict T2D better in women than in men: the ROC-AUC values were 0.66 ± 0.03 (P < 0.001) vs 0.59 ± 0.03 (P < 0.01) by systolic blood pressure and 0.60 ± 0.03 (P < 0.001) vs 0.54 ± 0.03 (ns) by diastolic blood pressure (Fig. 2C,F). Consistently, binary logistic regression modeling showed that both systolic and diastolic blood pressure were associated with T2D and the association was slightly stronger in women than in men (Table 1).

### Triglyceride, total cholesterol, and LDL predict T2D better in females than in males.

**Figure 3:**
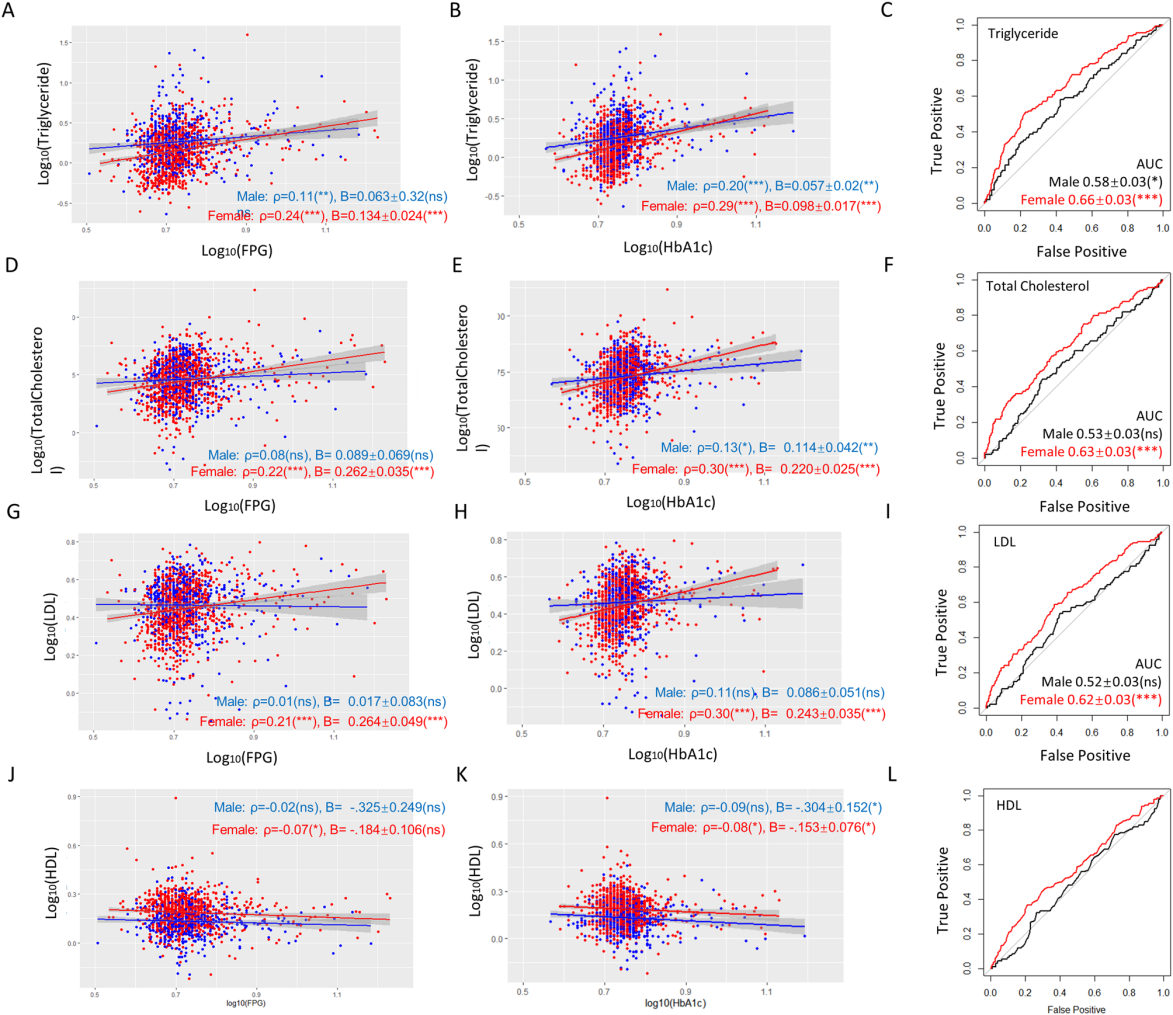
Triglyceride, total cholesterol, and LDL predict T2D better in women than in men. (**A**,**B**) Triglyceride has a tighter association (Spearman’s ρ) with FPG and HbA1c in women and affects FPG and HbA1c stronger (steeper slope) in women. P values, regression constant can be found in Tables S2 and S3. (**C**) Triglyceride is a stronger T2D predictor for women as judged by ROC-AUC analysis. (**D**,**E**) Total cholesterol levels tend to have tighter associations with FPG and HbA1c in women and affect FPG and HbA1c levels stronger in women. (**F**) Total cholesterol is a stronger T2D predictor for women. (**G**,**H**) LDL levels associate with FPG and HbA1c stronger in women and tend to affect FPG and HbA1c levels stronger in women. (**I**) LDL levels predict T2D in women but not men as judged by ROC-AUC analysis. (**J**,**K**) HDL has a very weak, negative correlation with FPG and HbA1c and shows no sex-difference. (**L**) HDL predicts T2D better in women than in men. *AUC* area under the curve, *ns* not significant, *P < 0.05, **P < 0.01, ***P < 0.001.

We next examined a panel of lipid profiles including triglyceride levels, total cholesterol, LDL, and HDL. Except for HDL, the lipid profiles were positively correlated to FPG and HbA1c levels (Fig. 3) and the correlations were stronger in women than in men for both FPG and HbA1c. Interestingly, after adjustment for age, smoking, overweight, hyperlipidemia, hyperuricemia, and T2D, no obvious sex difference was observed for above associations (Table S2). Except for HDL, the slopes of linear regression were steeper in women than in men, suggesting that changes in these lipid profiles affect FPG and HbA1c stronger in women than in men (Fig. 3).

All triglyceride levels, total cholesterol, LDL and HDL predicted T2D better in women than in men: the ROC-AUC values were 0.66 ± 0.03 (P < 0.001) vs 0.58 ± 0.03 (P < 0.05) by triglyceride, 0.63 ± 0.03 (P < 0.001) vs 0.53 ± 0.03 (ns), by total cholesterol, 0.62 ± 0.03 (P < 0.001) vs 0.52 ± 0.03 (P < 0.01) by LDL and 0.58 ± 0.03 (P < 0.01) vs 0.50 ± 0.03 (ns) by HDL (Fig. 3C,F,I,L). Significantly, binary logistic regression modeling suggested that except for HDL, increases in these lipid parameters were associated with T2D in women but not in men (Table 1). These remained true after adjustment for age, smoking, overweight, hyperlipidemia, hyperuricemia, and T2D. HDL was negatively associated with T2D and interestingly, women were benefited more than men by its increase. Together, the results suggest that triglyceride levels, total cholesterol, and LDL could predict T2D better in women than in men.

### Fasting insulin and proinsulin levels predict T2D better in females better in males

**Figure 4:**
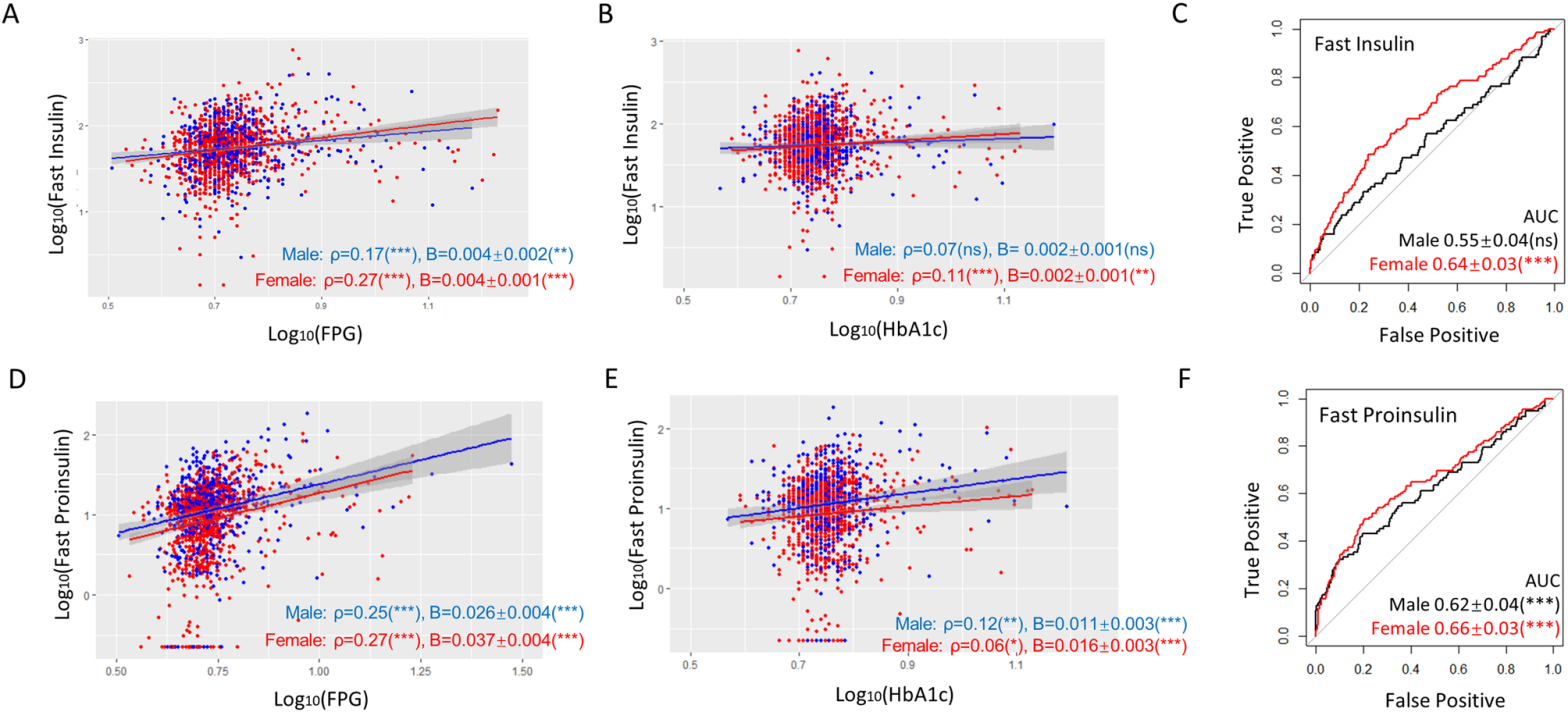
Fasting insulin and proinsulin levels predict T2D better in women than in men. (**A**,**B**) Fasting insulin levels associate with and affect FPG and HbA1c stronger in women than in men as judge from Spearman’s ρ and regression coefficient B. P values, regression constant can be found in Tables S2 and S3. (**C**) Fasting insulin is a stronger T2D predictor for women as judged by ROC-AUC values. (**D**) Fasting proinsulin levels associate with and affect FPG and HbA1c stronger in women than in men. (**E**) Fasting proinsulin levels are weakly associated with HbA1c in women. (**F**) Fasting proinsulin is a stronger T2D predictor for women as judged by ROC-AUC values. *AUC* area under the curve, *ns* not significant, **P < 0.01, ***P < 0.001.

The correlation of fasting insulin levels with FPG was higher (ρ = 0.27 vs 0.17, P < 0.001) in women than in men (Fig. 4A). Although not significant, the correlation of fasting insulin levels with HbA1c was trending higher in women than in men (Fig. 4B and Table S2). The slope of linear regression was trending steeper in women than in men (Fig. 4A,B). Fasting proinsulin levels showed a weak association with FPG and such association was slightly higher in women than in men (Fig. 4D,E). Consistently, the ROC-AUC values were higher in women than in men if predicting T2D by fasting insulin levels, and slightly higher in women than in men by fasting proinsulin (Fig. 4C,F). The difference in binary logistic regression was marginal but still trending steeper in women than in men (Table 1). Interestingly, despite the stronger predicting power in women, fasting proinsulin was correlated with HbA1c stronger in men than in women.

We also examined postprandial insulin and proinsulin levels and found no correlation to FPG and HbA1c (Figure S1 and Table S2). Linear regression was conducted and no significant difference between men and women was observed (Table S3). Consistently, no obvious sex difference was found in the prediction power as judged from the ROC-AUC values (Figure S1). Proinsulin/insulin ratio (P/I ratio) was not significantly associated with FPG or HbA1c in women and only a slight association was found in men (Table S2). Interestingly however, P/I ratio predicted T2D better in men than in women, although weakly (Figure S2). Similarly, the association and regression pattern of P/I ratio to FPG and HbA1c after 2 h of glucose stimulation was not different between men and women (Tables S2 and S3), consistent with the lack of sex difference and the weak power of postprandial P/I ratio in predicting T2D (Figure S2).

### Sex-difference in the cutoffs of predicting parameters individually or in combination

**Figure 5:**
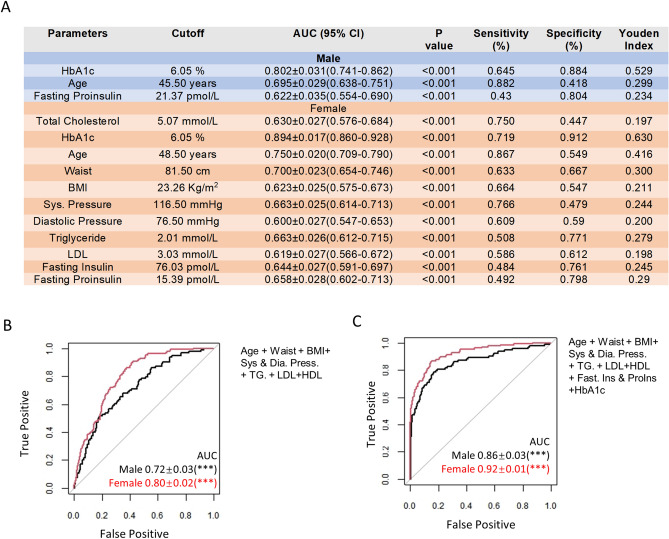
Sex-difference in the cutoffs of the predicting parameters individually or in combination. (**A**) The cutoffs of 3 parameters in men and 11 parameters in women having significant (P < 0.05) prediction power (ROC-AUC values > 0.6). A comprehensive comparison between men and women on the cutoffs, ROC-AUC values, sensitivity, specificity, and Youden Index is shown in Table S4. (**B**) A combination of age, waist, BMI, systolic and diastolic blood pressure, triglyceride, LDL, and HDL in T2D prediction by ROC analysis. (**C**) A combination of fasting insulin and proinsulin and HbA1c with parameters in (**C**) for T2D prediction. *AUC* area under the curve, ***P < 0.001.

Based on the results from ROC-AUC analysis, we calculated the optimal cutoffs for all parameters in predicting T2D using Youden’s J statistics and compared the sensitivity, specificity, and Youden Index between men and women (Table S4 and Fig. 5A). At the optimal cutoffs, women have a higher Youden Index for HbA1c, fasting and stimulated insulin and proinsulin, lipid profiles including total cholesterol, triglyceride and LDL, systolic and diastolic blood pressure, and physical parameters including age, waist, and BMI. Only 3 parameters (HbA1c, age, and fasting proinsulin) in men showed ROC-AUC values higher than the threshold value of 0.6, while there were 11 such parameters for women (Fig. 5A). We also calculated the ROC-AUC values for a combination of parameters that were preferentially more sensitive in women. Combining age, waist, BMI, systolic and diastolic blood pressure, triglyceride, LDL and HDL resulted in ROC-AUC values of 0.80 ± 0.02 (P < 0.001) in women vs 0.72 ± 0.03 (P < 0.001) in men (Fig. 5B). Further adding fasting insulin and proinsulin and HbA1c resulted in 0.92 ± 0.01 (P < 0.001) in women vs 0.86 ± 0.03 (P < 0.001) in men (Fig. 5C). Overall, by multiple parameters, T2D was more predictable in women than in men in our cohort study.

## Discussion

The sex difference in disease results from a combination of human genetics, physiology, behavior, or anatomy. It is believed that nearly all human diseases are sexually dimorphic, manifesting in prevalence, age of onset, severity, or disease course^23^. For example, osteoporosis, autoimmune diseases, and Alzheimer’s diseases are more prevalent in women^24–26^. Autism and some cancers such as stomach cancer, oesophageal cancer, and liver cancer have a higher prevalence in men^27^. Recently, the new concept of sex and gender medicine has begun to attract attention. This new concept proposes the separation of men and women in the diagnosis, prevention and treatment of diseases which are currently treated by ‘one-size-fits-all' approach, and believes that sex-based prevention measures and therapies would benefit patients of both genders^6^.

Few studies to date have focused on the sex difference in T2D, especially the diagnostic and predictive parameters^28^. In this study, we obtain a panel of the most common anthropometric and biochemical parameters from 1579 participants distributed across the Hainan Province, China, and systematically study the association with FPG, HbA1c and, T2D incidence. We focused on comparing the difference between males and females. Our results show that HbA1c, age, waist circumference, BMI, systolic and diastolic blood pressure, triglyceride, total cholesterol, LDL, HDL, fasting insulin, and proinsulin levels all predict T2D better in women than in men. To our knowledge, this is the first study of its kind systematically focusing on the sex difference in predicting T2D.

We have also examined vitamin D3 and uric acid levels. Higher vitamin D3 levels have been shown to correlate with a lower risk of T2D and proposed to be a useful predictor for T2D development^29–32^. The link between blood uric acid and T2D remains unclear^33–35^. Different from the above studies, our results show that the predicting powers of vitamin D3 and uric acid are weak (ROC-AUC < 0.6). No difference in the ROC-AUC values between women and men was observed (Figures S3 and S4). The discrepancy could be due to the cohort difference, including age, ethnicity, and eating habit. However, our results are in line with the failure in clinical trials of vitamin D supplements for improving glucose tolerance and insulin sensitivity^36^.

By analyzing multiple anthropometric and biochemical measures, we rank them by their ability to predict T2D according to the ROC-AUC values: in women, age (0.75) > waist circumference (0.70) > systolic pressure (0.66) = triglyceride (0.66) = fasting proinsulin (0.66) > fasting insulin (0.64) > total cholesterol (0.63) > BMI (0.62) = LDL (0.62) > LDL (0.62) > diastolic pressure (0.60); in men, age (0.69) > fasting proinsulin (0.62) > fasting P/I ratio (0.61). There are several interesting interpretations from this result. First, these measures are mostly poor predictors for men but moderate predictors for women, arguing for the importance of sex-based subgroup analysis in evaluating predictors for T2D. Second, different from many previous studies focusing only on one or two factors and proposing BMI and P/I ratio as good T2D predictors, our study finds them to be much poorer predictors than many other measures including blood pressure and lipid profiles. Third, in women, waist circumference alone rather than BMI is a good T2D predictor (ROC-AUC > 0.70), which is somehow surprising but consistent with two previous studies^37,38^.

In all, by thoroughly comparing the correlation, regression, and ROC analysis of common anthropometric and metabolic measures between men and women from a large cohort in Hainan China, we show that T2D is more readily predicted by these parameters in women than in man, suggesting the importance of weighing sex difference in T2D diagnosis and prediction.

## Methods

### Subjects

Subjects participating in this study are ethnically a combination of east Asian and southeast Asian distributing across cities and countryside of Hainan Province, China. This study includes 1579 men and women of different ages, sex across different socioeconomical status and education levels. Such information is collected based on a survey before admitting participants for the glucose tolerance test. The study was approved by the Ethical Committee of Hainan General Hospital and all participants gave written informed consent. All methods involving humans were carried out in accordance with relevant guidelines and regulations. Original data was published before^39^.

### Anthropometric and biochemical measurements

Weight and height were measured on a mechanic scale and a rod mounted on the wall. Values for weight and height were kept to the nearest 0.1 kg and 0.5 cm, respectively. BMI was calculated by dividing weight (kg) by the square of height (m^2^). Waist circumference was measured at the midpoint between the lateral iliac crest and lowest rib to the nearest 0.5 cm. Systolic pressure and diastolic pressure were obtained by a standard sphygmomanometer. Glucose stimulation was conducted by a standard protocol of a 2-h, 75-g oral glucose tolerance test (OGTT). Fasting was defined by no food uptake and drinks (except for water) for at least 10 h. Blood was then drawn at the fasting state and glucose-stimulated state. Blood glucose, HbA1c, triglyceride, total cholesterol, LDL, HDL, fasting insulin, and proinsulin were measured by standard protocols in the Medical Laboratory in Hainan General Hospital.

### Data analysis

Original data were published before^39^. Data of all 1579 participants were grouped by sex. Association/correlation study was conducted by calculating Spearman’s rank correlation coefficient using SPSS software. Linear regression and binary regression analysis were conducted by using SPSS software. ROC graph and AUC values were derived from pROC package in Rstudio. To visualize the linear regression, data were first converted by log10 to obtain normally distributed data, then visualized by Rstudio using ggplot2 package. A small number of outliers were removed for visualization purpose.

### Statistical significance

Statistical analysis was conducted using the default methods in IBM SPSS version 24. Briefly, Chi-square was used to compare the frequency of diabetic complications and other conditions including smoking between the men group and women group. A two-tailed, unpaired Student’s t-test was used to compared mean values of parameters. Association was analyzed by using the Spearman method. P < 0.05 was considered statistically significant.

## Supplementary Information

Supplementary Information
