# Supplementary material for: Type 2 diabetes is more predictable in women than men by multiple anthropometric and biochemical measures
Source: Sci Rep. 2021 Mar 15;11:6062. doi: 10.1038/s41598-021-85581-z (PMC7960723; doi:10.1038/s41598-021-85581-z)
Supplement: Supplementary file 1 — Supplementary Information [file 41598_2021_85581_MOESM1_ESM.docx]

Supplemental information

Type 2 diabetes is more predictable in women than men by multiple anthropometric and biochemical measures

Tangying Li^1^, Huibiao Quan^2,*^, Huachuan Zhang^3^, Leweihua Lin^2^, Lu Lin^2^, Qianying Ou^2^, Kaining Chen^2^

1. Department of Health Care Centre, Hainan General Hospital, Hainan Affiliated Hospital of Hainan Medical University, Haikou, Hainan 570311, China

2. Department of Endocrinology, Hainan General Hospital, Hainan Affiliated Hospital of Hainan Medical University, Haikou, Hainan 570311, China

3. Department of Endocrinology Laboratory, Hainan General Hospital, Hainan Affiliated Hospital of Hainan Medical University, Haikou, Hainan 570311, China

* Corresponding author

Huibiao Quan, MD

Department of Endocrinology, Hainan General Hospital, Hainan Affiliated Hospital of Hainan Medical University. No.19 Xiuhua Road, Haikou, Hainan 570311, China.

E-Mail: [qhb13876078153@hainmc.edu.cn](mailto:qhb13876078153@hainmc.edu.cn)

Tel: +86-0898-68636106

**Table S1**

**Physical and metabolic characteristics of 1579 participants in this study in Hainan Province, China.**

|  | *Male* | *Female* | *P value* |
| --- | --- | --- | --- |
| *Number of subjects* | 567 | 1012 | - |
| *Diabetes* | 93 | 128 | 0.039 |
| *Angina pectoris* | 3 | 4 | 0.71 |
| *Myocardial infarction* | 2 | 0 | 0.059 |
| *Dissecting aneurysm* | 0 | 1 | 0.454 |
| *heart failure* | 1 | 0 | 0.181 |
| *Cerebral hemorrhage* | 0 | 0 | - |
| *Cerebral infarction* | 3 | 2 | 0.261 |
| *Diabetic foot* | 1 | 0 | 0.181 |
| *arterial occlusive disease of low extremity* | 1 | 0 | 0.181 |
| *central retinal artery occlusion* | 0 | 0 | - |
| *Other diabetic complications* | 4 | 3 | 0.259 |
| *High blood pressure* | 87 | 131 | 0.185 |
| *Overweight/obese (BMI>25 Kg/m^2^)* | ***226*** | ***299*** | <0.001 |
| *Current and ex-smoker* | 290 | 21 | <0.001 |
| *Hyperlipidemia* | 104 | 128 | 0.002 |
| *Hyperuricemia* | 48 | 26 | <0.001 |
| *Iodine medicine* | ***10*** | ***35*** | ***0.052*** |
| *Diabetic medicine* | ***23*** | ***24*** | ***0.059*** |
| *Age(year)* | 49.16±13.03 | 47.43±13.5 | 0.013 |
| *Weight(Kg)* | 67.76±9.96 | 56.30±9.40 | <0.001 |
| *Height(cm)* | 166.84±6.10 | 155.17±6.07 | <0.001 |
| *Body mass index (BMI**, Kg/m^2^)* | 24.319±3.06 | 23.38±3.67 | <0.001 |
| *Waist circumference(cm)* | 86.25±8.64 | 78.52±9.16 | <0.001 |
| *Systolic pressure (mmHg)* | 128.26±18.00 | 121.99±20.15 | <0.001 |
| *Diastolic pressure(mmHg)* | 80.75±12.50 | 76.11±11.67 | <0.001 |
| *Heartbeat(times/min)* | 79.50±11.28 | 82.83±12.48 | <0.001 |
| *FPG(mmol/L)* | 5.56 ±1.70 | 5.43±1.28 | 0.107 |
| *OGTT2hPG(mmol/L)* | 8.04±3.68 | 7.67±3.19 | 0.043 |
| *Triglyceride(mmol/L)* | 2.27±2.21 | 1.71±1.69 | <0.001 |
| *Total cholesterol(mmol/L)* | 5.48±1.04 | 5.39±1.12 | 0.113 |
| *LDL(mmol/L)* | 3.05±0.86 | 2.90±0.82 | 0.001 |
| *HDL(mmol/L)* | 1.38±0.28 | 1.57±0.38 | <0.001 |
| *Blood uric acid(μmol/L)* | 419.24±80.42 | 320.13±73.19 | <0.001 |
| *HbA1c(%)* | 5.78±1.03 | 5.66±0.92 | 0.019 |
| *VitaminD3(ng/mL)* | 43.98±11.84 | 34.38±8.43 | <0.001 |
| *Fasting proinsulin(pmol/L)* | 17.13±17.18 | 12.17±9.82 | <0.001 |
| *2h proinsulin(pmol/L)* | 73.50±64.31 | 56.50±44.91 | <0.001 |
| *Fasting insulin(pmol/L)* | 66.82±47.17 | 66.13±51.57 | 0.785 |
| *2h insulin(pmol/L)* | 502.53±458.33 | 496.80±443.29 | 0.809 |

**Table S2**

**Spearman correlation coefficient in male and female by SPSS**

|  | Male | | | | Female | | | |
| --- | --- | --- | --- | --- | --- | --- | --- | --- |
|  | Non-adjusted | | Adjusted* | | Non-adjusted | | Adjusted* | |
|  | Rho | P | Rho | P | Rho | P value | Rho | P |
| HbA1c~FPG | **0.441** | **<0.001** | **0.268** | **<0.001** | **0.496** | **<0.001** | **0.274** | **<0.001** |
| Age~FPG | **0.304** | **<0.001** | **0.216** | **<0.001** | **0.394** | **<0.001** | **0.283** | **<0.001** |
| Age~HbA1c | **0.252** | **<0.001** | **0.195** | **<0.001** | **0.438** | **<0.001** | **0.347** | **<0.001** |
| Waist~FPG | **0.149** | **<0.001** | 0.03 | 0.472 | **0.310** | **<0.001** | **0.100** | **0.001** |
| Waist~HbA1c | **0.207** | **<0.001** | 0.082 | 0.052 | **0.305** | **<0.001** | 0.079 | 0.012 |
| BMI~FPG | **0.173** | **<0.001** | 0.083 | 0.051 | **0.268** | **<0.001** | **0.138** | **<0.001** |
| BMI~ HbA1c | **0.134** | **<0.001** | **0.085** | **0.045** | **0.233** | **<0.001** | **0.077** | **0.015** |
| Systolic Pressure~ FPG | **0.252** | **<0.001** | **0.164** | **<0.001** | **0.339** | **<0.001** | **0.159** | **<0.001** |
| Systolic Pressure~ HbA1c | **0.120** | **<0.001** | 0.018 | 0.665 | **0.293** | **<0.001** | **0.073** | **0.021** |
| Diastolic pressure ~ FPG | 0.052 | 0.219 |  |  | **0.224** | **<0.001** | **0.139** | **<0.001** |
| Diastolic pressure ~ HbA1c | **0.198** | **<0.001** | 0.010 | 0.815 | **0.119** | **<0.001** | 0.040 | 0.899 |
| Triglyceride ~FPG | 0.111 | 0.008 |  |  | **0.235** | **<0.001** | **0.082** | **0.010** |
| Triglyceride ~ HbA1c | **0.196** | **<0.001** | **0.162** | **<0.001** | **0.288** | **<0.001** | **0.150** | **<0.001** |
| Total Cholesterol ~FPG | 0.077 | 0.069 |  |  | **0.224** | **<0.001** | 0.060 | 0.060 |
| Total Cholesterol ~ HbA1c | **0.134** | **0.01** | **0.106** | **0.012** | **0.299** | **<0.001** | **0.137** | **<0.001** |
| LDL~FPG | 0.015 | 0.718 |  |  | **0.210** | **<0.001** | **0.067** | **0.034** |
| LDL~ HbA1c | 0.108 | 0.10 |  |  | **0.302** | **<0.001** | **0.171** | **<0.001** |
| HDL~FPG | -0.023 | 0.579 |  |  | **-.067** | **0.034** | -0.060 | 0.057 |
| HDL~ HbA1c | **-0.087** | **0.039** | **-0.084** | **0.047** | **-0.08** | **0.011** | **-0.085** | **0.007** |
| Fasting Insulin~ FPG | **0.173** | **<0.001** | **0.170** | **<0.001** | **0.266** | **<0.001** | **0.202** | **<0.001** |
| Fasting Insulin~ HbA1c | 0.069 | 0.101 |  |  | **0.114** | **<0.001** | 0.013 | 0.067 |
| Fasting Proinsulin~ FPG | **0.249** | **<0.001** | **0.210** | **<0.001** | **0.266** | **<0.001** | **0.193** | **<0.001** |
| Fasting Proinsulin~ HbA1c | **0.117** | **0.005** | 0.044 | 0.304 | **0.063** | **0.045** | -0.058 | 0.069 |
| Stimulated Insulin~ FPG | 0.051 | 0.225 |  |  | **0.152** | **<0.001** | **0.119** | **<0.001** |
| Stimulated Insulin~ HbA1c | 0.003 | 0.949 |  |  | 0.021 | 0.058 |  |  |
| Stimulated Proinsulin~ FPG | **0.112** | **0.008** | 0.080 | 0.060 | **0.176** | **<0.001** | **0.141** | **<0.001** |
| Stimulated Proinsulin~HbA1c | **-0.101** | **0.016** | **-0.166** | **<0.001** | **-0.092** | **0.004** | **-0.182** | **<0.001** |
| Fasting P/I ~FPG | **0.113** | **0.007** | 0.068 | 0.106 | 0.037 | 0.240 |  |  |
| Fasting P/I ~HbA1c | 0.055 | 0.191 |  |  | -0.048 | 0.128 |  |  |
| Stimulated P/I ~FPG | 0.044 | 0.291 |  |  | 0.038 | 0.232 |  |  |
| Stimulated P/I ~HbA1c | **-0.096** | **0.022** | **-0.112** | **0.008** | **-0.142** | **<0.001** | **-0.179** | **<0.001** |
| Vitamin D3 ~FPG | **0.096** | **0.023** | 0.081 | 0.055 | **0.180** | **<0.001** | **0.102** | **0.001** |
| Vitamin D3 ~HbA1c | -.0.052 | 0.221 |  |  | **0.086** | **0.006** | -0.019 | 0.541 |
| Blood Uric Acid ~FPG | -0.004 | 0.931 |  |  | **0.182** | **<0.001** | **0.102** | **0.001** |
| Blood Uric Acid ~HbA1c | -0.007 | 0.867 |  |  | **0.150** | **<0.001** | 0.056 | 0.079 |

* adjusted for age, smoking, overweight/obese (BMI>25), hyperuricemia, hyperlipidemia and diabetes.

**Table S3**

**Linear regression analysis in male and female by SPSS**

|  | | Male | |  | Female | |  |
| --- | --- | --- | --- | --- | --- | --- | --- |
|  | **Dependent variable** | **Constant** | **Coefficient (B)** | **P value** | **Constant** | **Coefficient (B)** | **P value** |
| HbA1c | FPG | -.536 | 1.054 | <0.001 | -0.845 | 1.108 | <0.001 |
| Age | FPG | 4.388 | 0.024 | <0.001 | 4.061 | 0.029 | <0.001 |
| Age | HbA1c | 5.236 | 0.011 | 0.001 | 4.630 | 0.022 | <0.001 |
| Waist | FPG | 4.643 | 0.011 | 0.197 | 2.790 | 0.034 | <0.001 |
| Waist | HbA1c | 4.358 | 0.017 | 0.001 | 3.781 | 0.024 | <0.001 |
| BMI | FPG | 5.051 | 0.021 | 0.364 | 4.157 | 0.055 | <0.001 |
| BMI | HbA1c | 4.997 | 0.032 | 0.023 | 4.777 | 0.038 | <0.001 |
| Systolic Pressure | FPG | 4.501 | 0.008 | 0.037 | 3.602 | 0.015 | <0.001 |
| Systolic Pressure | HbA1c | 5.463 | 0.003 | 0.297 | 4.440 | 0.010 | <0.001 |
| Diastolic pressure | FPG | 4.843 | 0.009 | 0.118 | 4.194 | 0.016 | <0.001 |
| Diastolic pressure | HbA1c | 5.758 | <0.001 | 0.916 | 5.119 | 0.007 | 0.004 |
| Triglyceride | FPG | 5.423 | 0.063 | 0.053 | 5.202 | 0.134 | <0.001 |
| Triglyceride | HbA1c | 5.658 | 0.057 | 0.004 | 5.496 | 0.098 | <0.001 |
| Total Cholesterol | FPG | 5.079 | 0.089 | 0.197 | 4.016 | 0.262 | <0.001 |
| Total Cholesterol | HbA1c | 5.163 | 0.114 | 0.006 | 4.480 | 0.220 | <0.001 |
| LDL | FPG | 5.512 | 0.017 | 0.834 | 4.665 | 0.264 | <0.001 |
| LDL | HbA1c | 5.526 | 0.086 | 0.090 | 4.959 | 0.243 | <0.001 |
| HDL | FPG | 6.014 | S | 0.193 | 5.722 | -0.184 | 0.084 |
| HDL | HbA1c | 6.208 | -0.304 | 0.046 | 5.905 | -0.153 | 0.046 |
| Fasting Insulin | FPG | 5.282 | 0.004 | 0.005 | 5.147 | 0.004 | <0.001 |
| Fasting Insulin | HbA1c | 5.682 | 0.002 | 0.089 | 5.564 | 0.002 | 0.007 |
| Fasting Proinsulin | FPG | 5.120 | 0.026 | <0.001 | 4.986 | 0.037 | <0.001 |
| Fasting Proinsulin | HbA1c | 5.603 | 0.011 | <0.001 | 5.469 | <0.001 | <0.001 |
| Stimulated Insulin | FPG | 5.683 | <0.001 | 0.133 | 5.450 | <0.001 | 0.698 |
| Stimulated Insulin | HbA1c | 5.868 | <0.001 | 0.094 | 5.709 | <0.001 | 0.172 |
| Stimulated Proinsulin | FPG | 5.507 | 0.001 | 0.474 | 5.336 | 0.002 | 0.058 |
| Stimulated Proinsulin | HbA1c | 5.833 | 0.001 | 0.360 | 5.750 | -0.002 | 0.020 |
| Fasting P/I | FPG | 4.984 | 2.063 | <0.001 | 5.391 | 0.186 | 0.227 |
| Fasting P/I | HbA1c | 5.513 | 0.975 | <0.001 | 5.650 | 0.064 | 0.565 |
| Stimulated P/I | FPG | 5.554 | 0.040 | 0.411 | 5.306 | 0.828 | 0.005 |
| Stimulated P/I | HbA1c | 5.784 | 0.012 | 0.690 | 5.651 | 0.088 | 0.678 |
| Vitamin D3 | FPG | 5.582 | <0.001 | 0.954 | 4.848 | 0.017 | <0.001 |
| Vitamin D3 | HbA1c | 6.026 | -0.005 | 0.145 | 5.517 | 0.004 | 0.212 |
| Blood Uric Acid | FPG | 6.342 | -0.002 | 0.038 | 5.144 | 0.001 | 0.103 |
| Blood Uric Acid | HbA1c | 6.210 | -0.001 | 0.065 | 5.592 | <0.001 | 0.567 |

**Table S4 ROC-AUC analysis and cutoff for diabetes prediction by different parameters.**

|  | **Sex** | **Cutoff** | **AUC (95% CI)** | **P value** | **Sensitivity (%)** | **Specificity (%)** | **Youden Index** |
| --- | --- | --- | --- | --- | --- | --- | --- |
| HbA1c(%) | M | 6.05 | 0.802±0.031(0.741-0.862) | <0.001 | **0.645** | **0.884** | **0.529** |
|  | F | 6.05 | 0.894±0.017(0.860-0.928) | <0.001 | **0.719** | **0.912** | **0.630** |
| FPG (mmol/L) | M | 5.83 | 0.904±0.021(0.862-0.945) | <0.001 | 0.806 | 0.905 | 0.711 |
|  | F | 5.73 | 0.903±0.018(0.868-0.938) | <0.001 | 0.820 | 0.883 | 0.703 |
| Age (Year) | M | 45.50 | 0.695±0.029(0.638-0.751) | <0.001 | 0.882 | 0.418 | **0.299** |
|  | F | 48.50 | 0.750±0.020(0.709-0.790) | <0.001 | 0.867 | 0.549 | **0.416** |
| Waist (cm) | M | 84.50 | 0.547±0.031(0.486-0.609) | 0.148 | 0.677 | 0.418 | **0.095** |
|  | F | 81.50 | 0.700±0.023(0.654-0.746) | <0.001 | 0.633 | 0.667 | **0.300** |
| BMI(Kg/m^2^) | M | 25.27 | 0.507±0.032(0.445-0.569) | 0.841 | 0.409 | 0.659 | **0.068** |
|  | F | 23.26 | 0.623±0.025(0.575-0.673) | <0.001 | 0.664 | 0.547 | **0.211** |
| Sys. Pressure (mmHg) | M | 128.50 | 0.593±0.034(0.527-0.659) | 0.005 | 0.613 | 0.591 | **0.204** |
|  | F | 116.50 | 0.663±0.025(0.614-0.713) | <0.001 | 0.766 | 0.479 | **0.244** |
| Dia. Pressure (mmHg) | M | 82.50 | 0.539±0.034(0.474-0.605) | 0.229 | **0.505** | **0.586** | **0.092** |
|  | F | 76.50 | 0.600±0.027(0.547-0.653) | <0.001 | **0.609** | **0.59** | **0.200** |
| Triglyceride (mmol/L) | M | 1.95 | 0.581±0.033(0.516-0.646) | 0.014 | 0.581 | 0.574 | **0.155** |
|  | F | 2.01 | 0.663±0.026(0.612-0.715) | <0.001 | 0.508 | 0.771 | **0.279** |
| Cholesterol (mmol/L) | M | 5.89 | 0.537±0.033(0.472-0.601) | 0.262 | 0.441 | 0.679 | **0.120** |
|  | F | 5.07 | 0.630±0.027(0.576-0.684) | <0.001 | 0.750 | 0.447 | **0.197** |
| LDL (mmol/L) | M | 3.19 | 0.516±0.034(0.451-0.582) | 0.619 | **0.538** | **0.581** | **0.119** |
|  | F | 3.03 | 0.619±0.027(0.566-0.672) | <0.001 | **0.586** | **0.612** | **0.198** |
| HDL (mmol/L) | M | 1.51 | 0.502±0.033(0.439-0.566) | 0.943 | 0.323 | 0.736 | **0.059** |
|  | F | 1.34 | 0.580±0.028(0.526-0.635) | 0.003 | 0.367 | 0.790 | **0.157** |
| Fast. Insulin (pmol/L) | M | 84.64 | 0.547±0.035(0.478-0.616) | 0.153 | 0.333 | 0.783 | **0.116** |
|  | F | 76.03 | 0.644±0.027(0.591-0.697) | <0.001 | 0.484 | 0.761 | **0.245** |
| Fast. Proinsulin (pmol/L) | M | 21.37 | 0.622±0.035(0.554-0.690) | <0.001 | 0.43 | 0.804 | **0.234** |
|  | F | 15.39 | 0.658±0.028(0.602-0.713) | <0.001 | 0.492 | 0.798 | **0.29** |
| 2h insulin (pmol/L) | M | 462.7 | 0.541±0.034(0.474-0.608) | 0.214 | 0.505 | 0.616 | **0.121** |
|  | F | 693.9 | 0.554±0.030(0.494-0.613) | 0.049 | 0.352 | 0.808 | **0.16** |
| 2h Proinsulin (pmol/L) | M | 67.01 | 0.573±0.033(0.509-0.638) | 0.025 | 0.559 | 0.586 | **0.145** |
|  | F | 59.81 | 0.583±0.029(0.527-0.639) | 0.002 | 0.531 | 0.649 | **0.18** |
| Fast. P/I Ratio | M | 0.48 | 0.612±0.034(0.544-0.679) | 0.001 | 0.269 | 0.918 | 0.187 |
|  | F | 0.33 | 0.530±0.028(0.476-0.584) | 0.265 | 0.258 | 0.836 | 0.094 |
| 2h P/I Ratio | M | 0.41 | 0.529±0.034(0.462-0.596) | 0.372 | 0.215 | 0.884 | 0.099 |
|  | F | 0.125 | 0.536±0.027(0.482-0.589) | 0.193 | 0.586 | 0.513 | 0.099 |
| Blood Uric Acid | M | 412.5 | 0.575±0.032(0.512-0.639) | 0.022 | 0.613 | 0.548 | 0.161 |
|  | F | 392.5 | 0.579±0.029(0.523-0.636) | 0.004 | 0.281 | 0.869 | 0.15 |
| Vitamin D3 (ng/mL) | M | 51.95 | 0.507±0.033(0.443-0.571) | 0.833 | 0.312 | 0.78 | 0.092 |
|  | F | 43.65 | 0.536±0.028(0.482-0.591) | 0.182 | 0.211 | 0.876 | 0.087 |
| Age+Waist+BMI+  Sys & Dia. Press.  + TG. + LDL+HDL | M | - | 0.719±0.028(0.665-0.774) | <0.001 | 0.516 | 0.817 | **0.333** |
|  | F | - | 0.802±0.018(0.768-0.837) | <0.001 | 0.898 | 0.589 | **0.487** |
| Age+Waist+BMI+  Sys & Dia. Press.  + TG. + LDL+HDL  +Fast.Ins&ProIns  +HbA1c | M | - | 0.859±0.025(0.810-0.908) | <0.001 | 0.785 | 0.831 | **0.616** |
|  | F | - | 0.924±0.014(0.897-0.950) | <0.001 | 0.867 | 0.849 | **0.716** |

**Supplemental Figures**


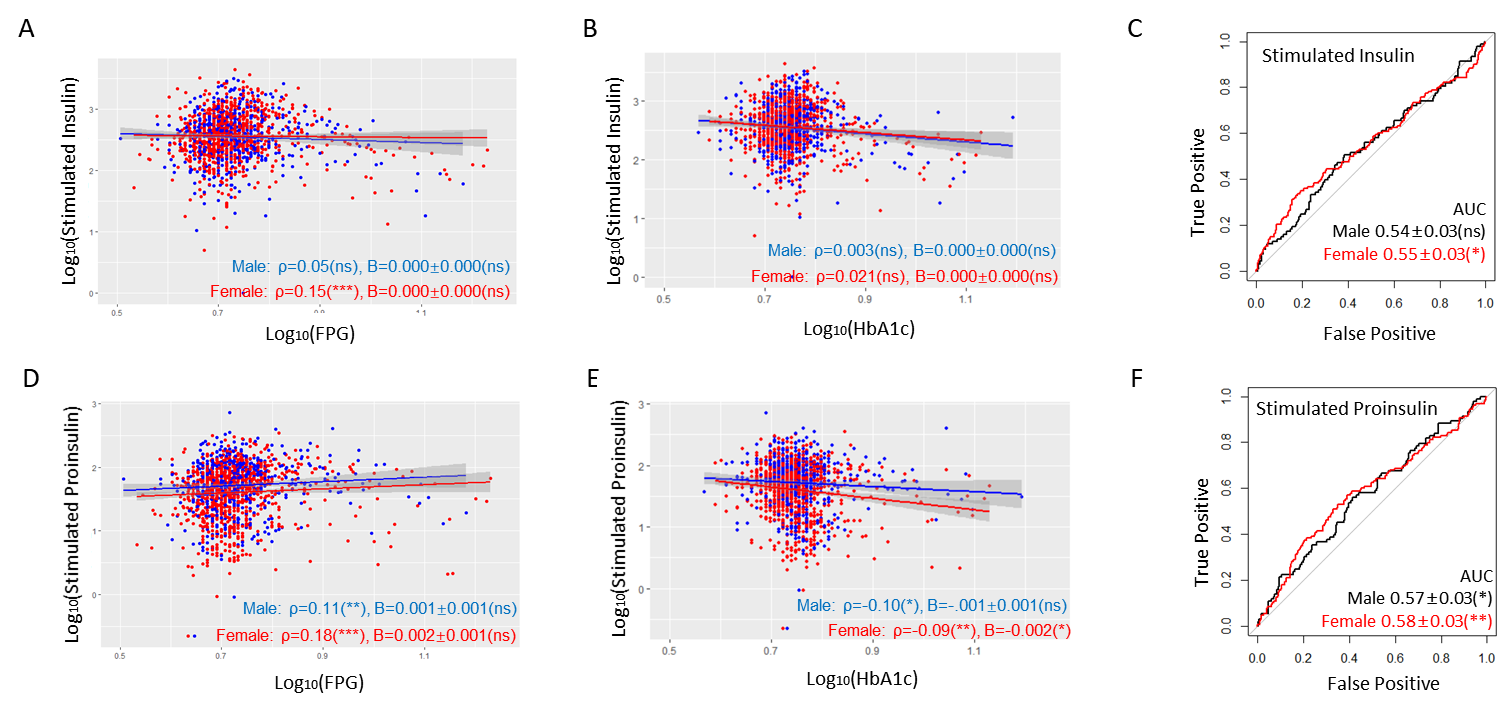


**Figure S1** Spearman’s correlation coefficient ρ and regression pattern of glucose-stimulated insulin to FPG (A) and HbA1c (B), and glucose-stimulated proinsulin to FPG (D) and HbA1c (E). ROC-AUC analysis showing that glucose-stimulated insulin (C) and proinsulin levels (F) are weak predictors for diabetes and have no difference between males and females. AUC values were presented with Standard Deviation (SD). *P<0.05, **P<0.01.


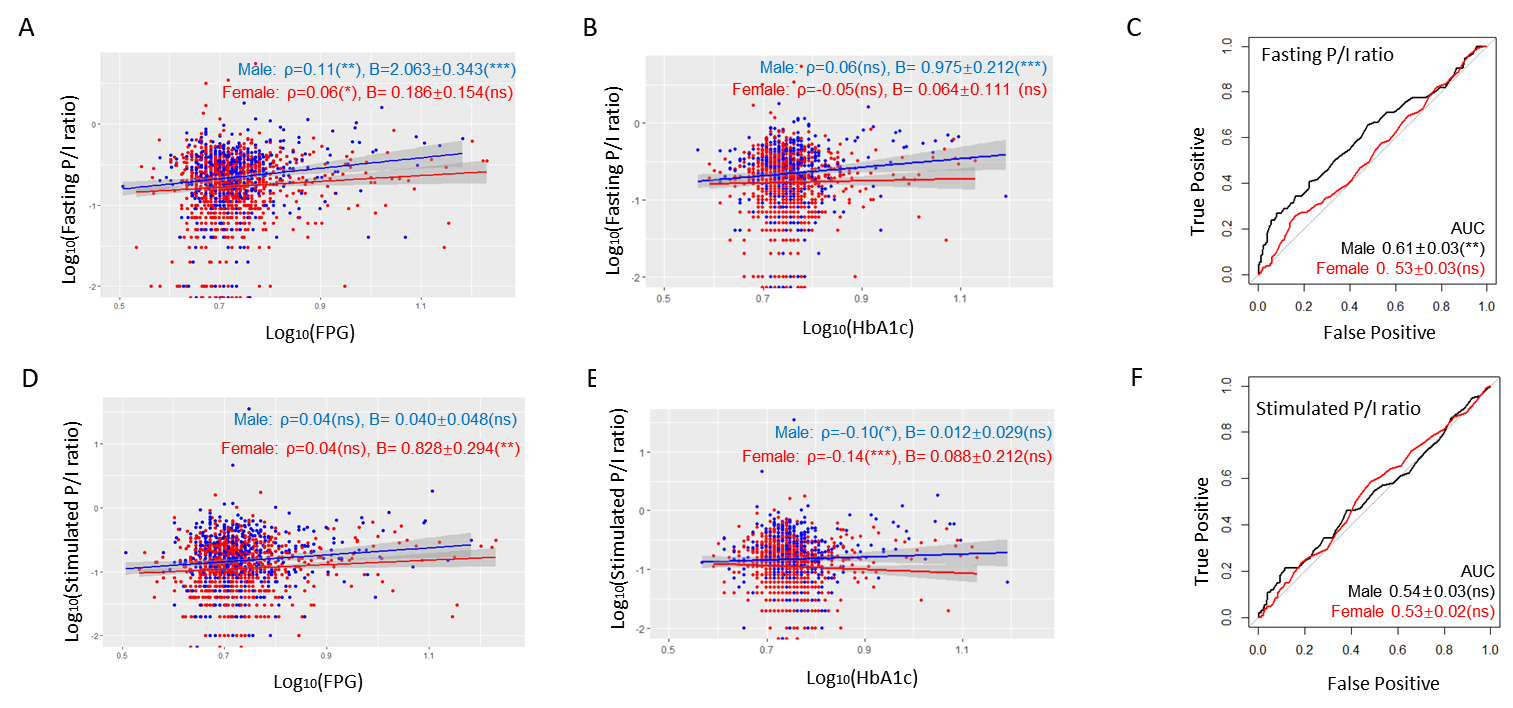


**Figure S2** Spearman’s correlation coefficient ρ and regression pattern of fasting P/I ratio to FPG (A) and HbA1c (B), and glucose-stimulated P/I ratio to FPG (D) and HbA1c (E). ROC-AUC analysis showing that fasting P/I ratio (C) predicts diabetes better in men and that glucose-stimulated P/I ratio (F) does not pass the threshold (AUC≥0.6) for predicting diabetes and has no sex-difference. AUC values were presented with Standard Deviation (SD). *P<0.05, **P<0.01, ***P<0.001, ns, not significant.


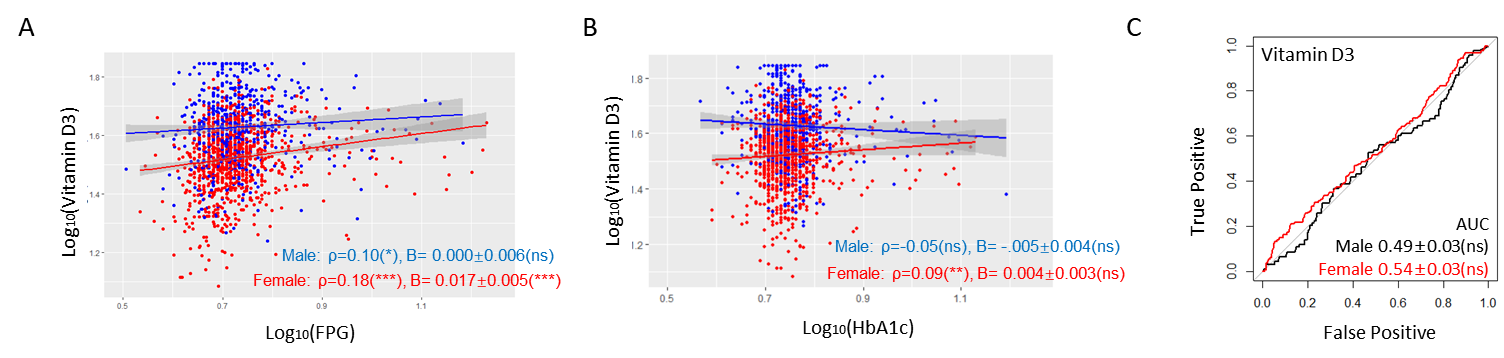


**Figure S3** Spearman’s correlation coefficient ρ and regression pattern of vitamin D3 to FPG (A) and HbA1c (B). ROC-AUC analysis showing vitamin D3 does not pass the threshold (AUC≥0.6) for predicting diabetes and have no sex-difference (C). AUC values were presented with Standard Deviation (SD). ***P<0.001, ns, not significant.


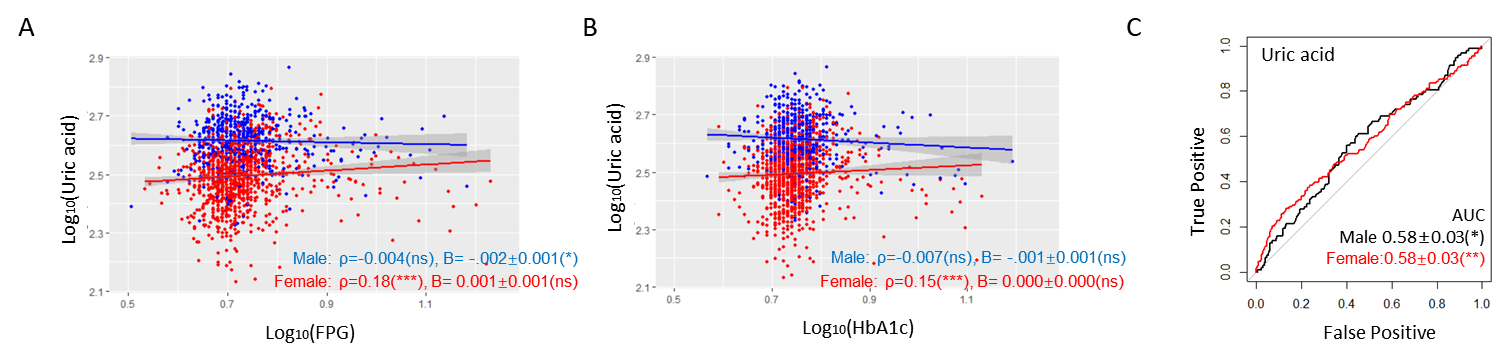


**Figure S4** Spearman’s correlation coefficient ρ and regression pattern of uric acid to FPG (A) and HbA1c (B). ROC-AUC analysis showing uric acid does not pass the threshold (AUC≥0.6) for predicting diabetes and have no difference between men and women (C). AUC values were presented with Standard Deviation (SD). *P<0.05, ns, not significant.
